# International external quality assessment for SARS-CoV-2 molecular detection and survey on clinical laboratory preparedness during the COVID-19 pandemic, April/May 2020

**DOI:** 10.2807/1560-7917.ES.2020.25.27.2001223

**Published:** 2020-07-09

**Authors:** Veerle Matheeussen, Victor M Corman, Oliver Donoso Mantke, Elaine McCulloch, Christine Lammens, Herman Goossens, Daniela Niemeyer, Paul S Wallace, Paul Klapper, Hubert GM Niesters, Christian Drosten, Margareta Ieven, on behalf of the RECOVER project and collaborating networks

**Affiliations:** 1 Department of Medical Microbiology, Vaccine and Infectious Disease Institute (VAXINFECTIO), University of Antwerp, Wilrijk, Belgium; 2 National Consultant Laboratory for Coronaviruses, Institute of Virology, Charité-Universitätsmedizin Berlin, Berlin, Germany; 3 Quality Control for Molecular Diagnostics (QCMD), Glasgow, United Kingdom; 4 School of Biological Sciences, Division of Infection, Immunity and Respiratory Medicine, The University of Manchester, Manchester, United Kingdom; 5 Division of Clinical Virology, Department of Medical Microbiology, University Medical Center Groningen, Groningen, the Netherlands; 6 These authors contributed equally to this work and share first authorship; 7 Details on these projects are noted in the Acknowledgements

**Keywords:** SARS-CoV-2, COVID-19, novel coronavirus, outbreak control, laboratory testing, molecular detection, external quality assessment (EQA), laboratory preparedness

## Abstract

Laboratory preparedness with quality-assured diagnostic assays is essential for controlling the current coronavirus disease (COVID-19) outbreak. We conducted an external quality assessment study with inactivated severe acute respiratory syndrome coronavirus 2 (SARS-CoV-2) samples to support clinical laboratories with a proficiency testing option for molecular assays. To analyse SARS-CoV-2 testing performance, we used an online questionnaire developed for the European Union project RECOVER to assess molecular testing capacities in clinical diagnostic laboratories.

Extensive laboratory testing is a prerequisite in containing and mitigating the impact of the ongoing coronavirus disease (COVID-19) pandemic while neither vaccine nor treatment are available [1]. Access to reliable diagnostic assays and adequate testing capacity for the causing severe acute respiratory syndrome coronavirus 2 (SARS-CoV-2) is essential for preparedness and response strategies worldwide [2]. Molecular methods such as RT-PCR are fundamental to early SARS-CoV-2 detection in suspected cases. Many in-house and commercial assays have been developed rapidly [3,4]. However, the quality and diagnostic performance of these tests have not been adequately validated; the World Health Organization (WHO) has therefore encouraged laboratories to participate in external quality assessment (EQA) schemes for this novel virus [5]. Here, we present the results of a first EQA on molecular detection of SARS-CoV-2 introduced by Quality Control for Molecular Diagnostics (QCMD, Glasgow, Scotland), an independent International EQA organisation. We also present data from a survey by the European Union (EU) project RECOVER, a COVID-19-related project originating from the EU-initiative Platform for European Preparedness Against (Re-)emerging Epidemics (PREPARE), assessing the molecular testing capacity and throughput of clinical laboratories in 36 countries.

## Coronavirus outbreak preparedness EQA pilot study

Between 6 April and 20 May 2020, each participating laboratory received a blinded panel of eight samples, with five samples containing serial 10-fold dilutions (2.30–5.30 dPCR log_10_ RNA copies/mL, one duplicated) of non-infectious SARS-CoV-2-positive supernatant obtained from Vero cell culture, two samples with cell culture-derived common human coronavirus (HCoV-NL63, HCoV-OC43) and one negative control with transport medium only. Supernatants were clarified by centrifugation at 300 × G for 10 min. In case of SARS-CoV-2, the material was heated (65 °C, 4 h) and gamma-irradiated (30 kGy) for inactivation. The 1.0-mL samples were pretested using the E gene assay (modified from [6]), and quantified by droplet digital PCR (Bio-Rad, Hercules, United States (US)). The EQA panels were shipped on dry ice. Participants were asked to treat and test the material like clinical samples using their routine molecular assay workflows and to report their results together with workflow details through a dedicated online reporting system. Laboratories submitted one dataset per applied workflow with extraction and amplification method used to test the provided samples.

In this first EQA round, 365 of 406 registered laboratories from 36 countries (25 European, nine Australian-Asian and two North American) submitted a total of 521 datasets with qualitative results. All participants received a rapid report after submission of their results, followed by an EQA report with individual performance and peer group assessment on completion of the study. The panel composition and overall performance per sample are shown in Table 1.

**Table 1:** Coronavirus disease (COVID-19) outbreak preparedness EQA pilot study, panel composition and overall performance per sample, April/May 2020 (n =521)

Sample code	Sample content^a^	Viral RNA concentration^b^ (dPCR log_10_ copies/mL)	Sample status^c^	Percentage correct qualitative results (all)		Reported Cq values (For information purposes only)	
				%	Total datasets	Median (range)	Total datasets
CVOP20S-01	SARS-CoV-2	4.30	CORE	98.1	521	29.1 (15.8–41.6)	449
CVOP20S-02	HCoV-NL63	4.64	EDUC	96.9	521	NA	NA
CVOP20S-03	SARS-CoV-2	3.30	CORE	96.9	521	32.2 (18.0–43.0)	450
CVOP20S-04	HCoV-OC43	4.03	EDUC	97.1	521	NA	NA
CVOP20S-05	Negative	NA	CORE	97.3	521	NA	NA
CVOP20S-06	SARS-CoV-2	4.30	CORE	98.5	521	29.2 (16.6–40.0)	450
CVOP20S-07	SARS-CoV-2	5.30	CORE	99.2	521	25.9 (12.0–39.0)	453
CVOP20S-08	SARS-CoV-2	2.30	CORE	86.0	521	35.0 (22.7–43.3)	400

Cq: quantification cycle; CVOP: Coronavirus outbreak preparedness; dPCR: digital PCR; EQA: external quality assessment; HCoV: human coronavirus: NA: not applicable; SARS-CoV-2: severe acute respiratory syndrome coronavirus 2.

^a^ Content of the EQA samples: SARS-CoV-2 strain BetaCoV/Munich/ChVir984/2020 provided by the Charité-Universitätsmedizin Berlin Institute of Virology, Berlin, Germany; human coronaviruses HCoV-NL63 and HCoV-OC43 cultivated by the University Medical Center Groningen, Groningen, the Netherlands, and sample specifications selected based on past performance data from regular provided coronavirus EQA schemes; negative control sample contained transport medium only (which was used as sample matrix for the EQA samples).

^b^ Values obtained using a dPCR assay (modified from [6]). Samples CVOP20S-07, -01, -03 and - 08 are in a calibrated dilution series. CVOP20S-06 is a duplicate sample of CVOP20S-01. The values provided are for reference only and have been established to support the consistency and traceability of the EQA materials as well as comparison of results across participating laboratories.

^c^ EQA samples are defined as ‘CORE’ or ‘EDUC’ (educational). Core proficiency samples are reviewed by scientific experts, based on scientific information, clinical relevance, current literature and where appropriate, professional clinical guidelines. Participating laboratories were expected to report the core proficiency samples (here: all SARS-CoV-2-positive samples and the negative control) correctly within the EQA scheme. Samples CVOP20S-02 and CVOP20S-04 were included in the panel as educational specificity samples and were expected to be reported as SARS-CoV-2 negative. Participants were not expected to report on the identity of the common human coronaviruses HCoV-NL63 or HCoV-OC43.

Detecting all SARS-CoV-2-positive samples and the negative sample correctly was defined as an acceptable level of proficiency. All core samples were correctly reported by 86.3% (315/365) of participating laboratories and in 83.1% (433/521) of datasets. When including the results reported for the two educational specificity samples, the overall percentage of correct results was 84.7% (309/365) on a laboratory level and 81.8% (426/521) for datasets. Incorrect results (including false-positive or not determined as laboratories should be able to report clear results by molecular methods) were reported for the true negative sample CVOP20S-05 in 14 (2.7%) datasets (with three false-positive and 11 not determined), for the SARS-CoV-2 negative educational sample CVOP20S-02 in 16 (3.1%) datasets (five false-positive, 11 not determined), and for the other SARS-CoV-2 negative educational sample CVOP20S-04 in 15 (2.9%) datasets (three false-positive, 12 not determined). At the laboratory level, 10 laboratories showed specificity issues by reporting false-positive or indeterminate results for the negative control and/or the two educational specificity samples, while 41 laboratories did not detect one or both of the two low-concentration samples (CVOP20S-08 and -03). Three laboratories showed both sensitivity and specificity issues and another two did not reproduce the results for the duplicated samples (CVOP20S-01 and -06).

Many participants provided quantification cycle (Cq) values in this EQA scheme. An overall median and range of the Cq values reported is shown for each SARS-CoV-2 positive sample in Table 1. While the medians reflect the expected gradation according to the virus concentration in the 10-fold sample dilutions, we observed considerable spread in Cq values with regard to the underlying target genes. This reflects differences in assay methods and the current lack of standardisation between assays. However, the overall percentage of correctly reported qualitative results was 98.3% for the E gene (reported in 197 datasets), 93.3% for the N gene (91 datasets), 96.3% for the ORF1ab gene (88 datasets), 95.6% for the RdRP gene (83 datasets) and 93.9% for the S gene (47 datasets) when used as the respective main molecular target (15 datasets did not state the target gene).

Logistic regression statistics, performed in SAS version 9.4 (SAS Institute Inc., Cary, US), was used to assess whether common technical factors, such as the extraction and amplification groups (by methods) listed in Table 2, influenced participant performance. Both extraction and amplification method were statistically significantly associated with correct classification of all samples (p < 0.001 for both). While the overall percentage of correct results was above 90% for each analysed group, certain groups showed statistically significant differences compared with the corresponding reference level, namely ‘any other extraction method’ or ‘any real-time in-house PCR’. As an example, for one fully automated approach (Certest VIASURE SARS-CoV-2 S gene real-time PCR kit (Certest, Zaragoza, Spain) used with the BD MAX system (Becton, Dickinson and Company, Franklin Lakes, US)) combining RNA extraction and subsequent real-time PCR (with only 90.3% and 90.8% overall correct results, respectively), all 14 false-negative results reported by 13 laboratories could be clearly linked to sensitivity issues with the two low-concentration samples in the panel. However, some of the observed differences for the groups listed in Table 2 could also be due to improper handling of the assays and/or the samples, since laboratories using the same assays showed large variations in their reported qualitative results (e.g. because of differences in the parameters used for indeterminate results).

**Table 2:** Factors influencing the performance of Coronavirus disease (COVID-19) outbreak preparedness EQA participants, logistic regressions on combined responses reported for all samples in the panel, April/May 2020 (n = 4,168)

Possible technical influence factors (grouped by methods)	Total responses per group	Percentage correct results		p value	Odds ratio	95% confidence interval
		%	Responses (n)			
Extraction method (overall)	4,168	96.3	4,012	< 0.001*		
Any other method (incl. seven in-house)	1,152	94.6	1,090	Reference level		
Abbott m2000sp	168	99.4	167	0.026*	9.50	1.31–68.97
BD MAX ExK TNA-3	144	90.3	130	0.040*	0.53	0.29–0.97
Roche Cobas Omni	304	99.3	302	0.003*	8.59	2.09–35.32
PSS MagDEA Dx	72	100	72	0.974	NA	NA
Roche MagNA Pure 96 DNA and Viral	336	96.7	325	0.119	1.68	0.88–3.23
Promega Maxwell RSC (Viral/Blood)	176	95.5	168	0.644	1.19	0.56–2.54
BioMérieux NucliSENS easyMAG	544	98.4	535	< 0.001*	3.38	1.67–6.86
Qiagen QIAamp Viral RNA Mini Kit	184	98.9	182	0.023*	5.18	1.26–21.35
Qiagen QIAsymphony DSP Virus	144	97.2	140	0.189	1.99	0.71–5.56
Seegene STARMag Universal Cart.	360	98.1	353	0.009*	2.87	1.30–6.32
Cepheid Xpert Xpress SARS-CoV-2	376	94.4	355	0.880	0.96	0.58–1.60
Not specified	208	92.8	193	0.295	0.73	0.41–1.31
Amplification method (overall)	4,168	96.3	4,012	< 0.001*		
Any real-time in-house PCR	952	96.7	921	Reference level		
Abbott RealTime SARS-CoV-2	136	99.3	135	0.138	4.54	0.62–33.53
Altona RealStar SARS-CoV-2 RT-PCR	224	99.1	222	0.072	3.74	0.89–15.73
Cepheid Xpert Xpress SARS-CoV-2	408	94.9	387	0.098	0.62	0.35–1.09
Certest VIASURE SARS-CoV-2	136	91.9	125	0.008*	0.38	0.19–0.78
Certest VIASURE SARS-CoV-2 S gene	152	90.8	138	< 0.001*	0.33	0.17–0.64
Elitech GeneFinder COVID-19 Plus	200	95.0	190	0.230	0.64	0.31–1.33
Roche cobas SARSCoV2	296	99.3	294	0.029*	4.94	1.18–20.77
Seegene Allplex 2019-nCoV	552	98.9	546	0.013*	3.06	1.27–7.39
TIB MOLBIOL LightMix Modular	216	98.6	213	0.153	2.39	0.72–7.89
Any other commercial test kit	896	93.9	841	0.004*	0.52	0.33–0.81

NA: not applicable.

* Statistically significant; p ≤ 0.05 (for details see text).

## Discussion

The data from this EQA study provide a comparison of available molecular assays for SARS-CoV-2 detection. About 23% of the used assays were in-house assays which performed at least as well as commercial ones or even better. The overall qualitative performance of the participating laboratories was at an acceptable level and showed similar rates of successful participation as compared with the molecular EQA for SARS (provided by WHO/European Network for Diagnostics of "Imported" Viral Diseases (ENIVD)) during the outbreak in 2002 and 2003, with similar requirements and rapid developments for diagnostics, where 51 of 58 laboratories from 38 countries achieved an acceptable level of proficiency [7]. Our EQA pilot study presented here revealed that analytical sensitivity and specificity remained variable.

Procedures should be reviewed where false-positive or indeterminate results have been reported which may lead to misdiagnosis and affect clinical decision making. As highly sensitive methods are required for early COVID-19 diagnostic screening, two low-concentration samples were included in the EQA panel close to the detection limit of published or commercial assays [3,4,8-10]. Laboratories that were unable to detect low-concentration samples, or whose methods showed Cq values greatly different from the provided medians, should strive to improve the sensitivity of their molecular assays to prevent false-negative results in respiratory samples with low viral concentrations from SARS-CoV-2-infected patients, e.g. during the early phase of infection.

## Survey on preparedness of diagnostic laboratories for SARS-CoV-2 detection

In parallel with the EQA study, laboratories were surveyed to assess their challenges in implementing and executing molecular testing capacity and throughput for SARS-CoV-2 detection, also in April and May 2020. Of the 365 laboratories that participated in our EQA pilot study, five were excluded from the survey as they were engaged in assay development rather than in routine SARS-CoV-2 detection.

Almost 80% of the participating laboratories (n = 360) are capable of generating a SARS-CoV-2 PCR result within 24 h after receiving the sample (Supplementary Figure S1). This turnaround time is comparable to the results of a smaller survey (n = 87 laboratories) performed earlier in the COVID-19 pandemic [11]. The daily test capacity, however, was much higher, with 48% of the laboratories capable of analysing more than 250 samples per day, while only 41% in the previous survey could handle more than 50 samples [11]. More than 70% of the laboratories indicated that collaboration with reference laboratories and (inter)national public health institutes has helped in establishing SARS-CoV-2 testing capability and capacity. However, challenges in implementation and execution of the testing remain, with lack of personnel/time and equipment being the predominant ones (Supplementary Figure S2). Compared with the earlier survey, challenges did shift over time, with lack of funding, an authorised mandate or unavailability of positive control material and/or a specificity panel becoming less important barriers.

## Conclusions

Molecular testing capacity and throughput on clinical diagnostic level have been rapidly implemented and should be supported by proficiency testing panels. EQA schemes, such as the one presented here, are a good opportunity for laboratories to assess the performance of their assays against international peer groups in line with agreed clinical practice, based on well-characterised samples, to identify any weaknesses with their procedures or methods. Laboratories should be aware of the limitations of their assays and perform their own validation and verification in line with ISO 15189 or equivalent requirements. Also, EQA data can provide valuable information for post-market surveillance of commercial assays, which is of particular importance in outbreak situations where data about the relative performance of methods are still limited and further clinical evaluations are ongoing. This way, the quality of COVID-19 diagnostic testing can be continuously ensured. Regional-specific EQA studies can also be done, but require a different approach in order to ensure comparability. For 2020, two further EQA programmes are planned by QCMD: a follow-up SARS-CoV-2 EQA study and a respiratory EQA scheme comprising SARS-CoV-2 and other respiratory pathogens [12].

## Supplementary Data

135000 Supplement
