# Supplementary material for: International external quality assessment for SARS-CoV-2 molecular detection and survey on clinical laboratory preparedness during the COVID-19 pandemic, April/May 2020
Source: Euro Surveill. 2020 Jul 9;25(27):2001223. doi: 10.2807/1560-7917.ES.2020.25.27.2001223 (PMC7364759; doi:10.2807/1560-7917.ES.2020.25.27.2001223)
Supplement: Supplement [file 20-201223_DONOSO-MANTKE_Supplement.pdf]

Supplementary figures, Survey on Preparedness of Diagnostic Laboratories for SARS-CoV-2 Detection, April/May 2020

This supplementary material is hosted by Eurosurveillance as supporting information alongside the article „International external quality assessment for SARS-CoV-2 molecular detection and survey on clinical laboratory preparedness during the COVID-19 pandemic, April/May 2020"on behalf of the authors who remain responsible for the accuracy and appropriateness of the content. The same standards for ethics, copyright, attributions and permissions as for the article apply. Supplements are not edited by Eurosurveillance and the journal is not responsible for the maintenance of any links or email addresses provided therein.

Supplementary Figure S1
Turnaround time (TAT), daily test capacity and collaboration for SARS-CoV-2 detection in participating laboratories, April/May 2020 (n= 360)

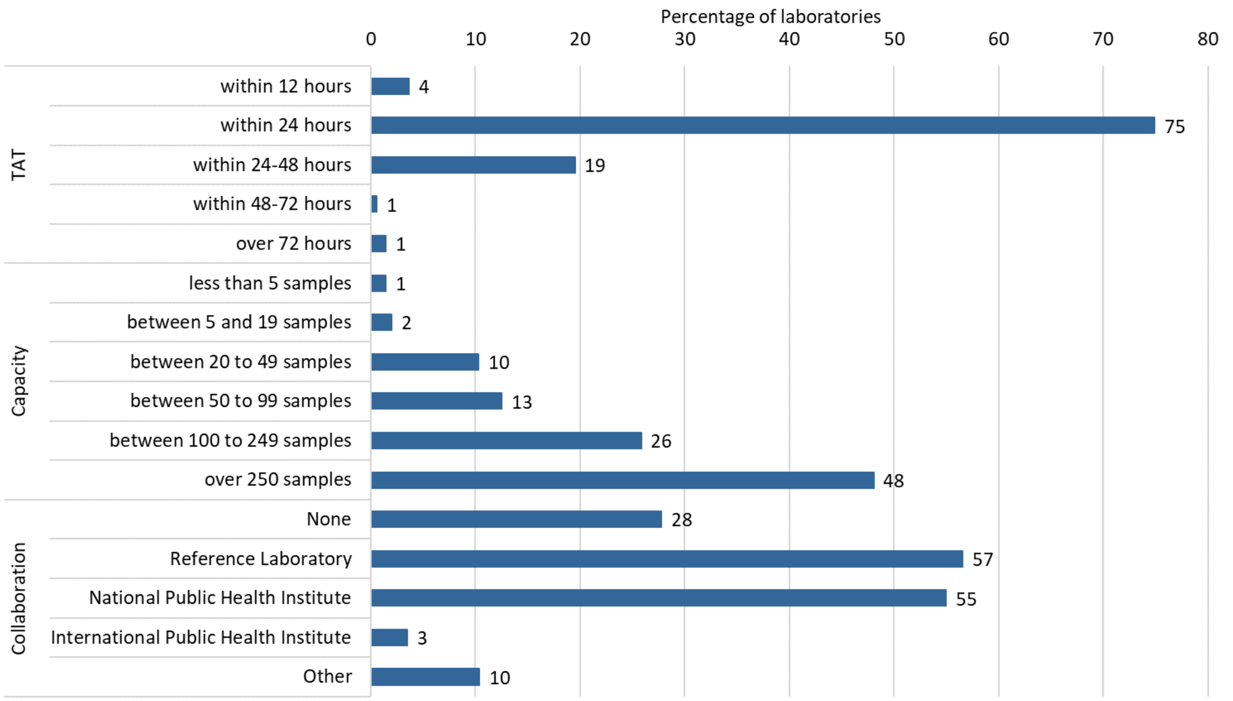

Supplementary Figure S2
Main challenges for implementation/execution of a SARS-CoV-2 molecular testing in participating laboratories, April/May 2020 (n= 360)

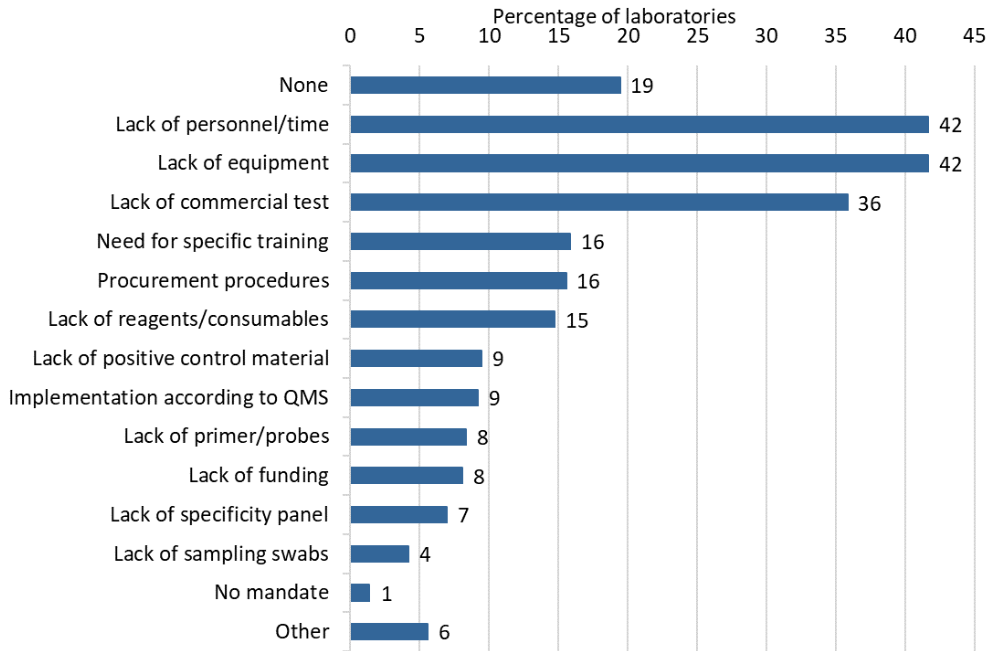

QMS: Quality Management System
